# Comparing computed tomography localization with daily ultrasound during image‐guided radiation therapy for the treatment of prostate cancer: a prospective evaluation

**DOI:** 10.1120/jacmp.v8i3.2268

**Published:** 2007-07-23

**Authors:** Steven J. Feigenberg, Kamen Paskalev, Shawn McNeeley, Eric M. Horwitz, Andre Konski, Lu Wang, Charlie Ma, Alan Pollack

**Affiliations:** ^1^ Fox Chase Cancer Center Department of Radiation Oncology Philadelphia Pennsylvania U.S.A.

**Keywords:** image‐guided radiotherapy, prostate cancer, CT localization, ultrasound localization

## Abstract

In the present paper, we describe the results of a prospective trial that compared isocenter shifts produced by BAT Ultrasound (Nomos, Sewicky, PA) to those produced by a computed tomography (CT) unit in the treatment room to aid in positioning during image‐guided radiation therapy.

The trial included 15 consecutive patients with localized prostate cancer. All patients underwent CT and MR simulation immobilized supine in an Alpha Cradle and were treated with intensity‐modulated radiation therapy. BAT Ultrasound was used daily to correct for interfraction motion by obtaining shift in the x, y, and z directions. Two days per week during therapy, CT scans blinded to the ultrasound shifts were obtained and recorded.

We analyzed 218 alignments from the 15 patients and observed a high level of correlation between the CT and ultrasound isocenter shifts (correlation coefficients: 0.877 anterior–posterior, 0.842 lateral, and 0.831 superior–inferior). The systematic differences were less than 1 mm, and the random differences were approximately 2 mm. The average absolute differences, including both systemic and random differences, were less than 2 mm in all directions.

The isocenter shifts generated by using a CT unit in the treatment room correlate highly with shifts produced by the BAT Ultrasound system.

PACS numbers: 87.53, 87.59.fm, 87.63.Df

## I. INTRODUCTION

Improvements in radiotherapy treatment and delivery techniques have led to improvements in treatment outcomes. Perhaps the best example is in the radiotherapeutic management of prostate cancer. Recently, a randomized study^(1)^ demonstrated that, as compared with conventional radiotherapy to 70 Gy, treatment with 78 Gy using conformal techniques led to a significant improvement in biochemical control.

As doses are increased, the precision and accuracy of dose delivery becomes crucial. However, daily uncertainties regarding patient setup reproducibility and the position of the prostate within the pelvis because of combinations of rectal and bladder filling diminish the ability to achieve this goal.^(2)^ The frequently changing nature of these variations has in the past made correction difficult and dictated that a wider margin of normal tissue be included within the radiation field to ensure complete tumor coverage.

In the treatment of prostate cancer, portal films are compared to simulation films to verify patient setup based on displacement of the bony pelvis. Unfortunately, the prostate can move in relation to bony anatomy. A recent study described the margins required to account for prostate motion 95% of the time: 5.3 mm in the lateral direction; 10.4 mm in the anterior–posterior (AP); and 10.4 mm in the superior–inferior.^(^ ^1^ ^,^ ^2^ ^)^ These margins related to interfraction setup error can be reduced by several techniques that directly visualize the prostate before treatment.

To reduce these uncertainties at Fox Chase Cancer Center in the mid‐1990s, we first performed daily computed tomography (CT) scans for patients receiving three‐dimensional conformal radiation therapy.^(3)^ In this costly and time‐consuming process, patients underwent CT simulation in one room and were then transported to the treatment machine while remaining in the immobilization cast.

A subsequent study at Fox Chase Cancer Center directly compared CT localization with an ultrasound system [BAT (B‐mode Acquisition and Targeting) Ultrasound: Nomos, Sewicky, PA] in the CT simulation room. That study demonstrated the equivalence of CT and ultrasound, but more importantly, demonstrated that ultrasound was simple and quick as compared with daily CT.^(^ ^4^ ^,^ ^5^ ^)^

Currently, we using the BAT Ultrasound system to obtain a daily ultrasound scan for every patient with prostate cancer treated at Fox Chase Cancer Center. Unfortunately, to be reliable, the BAT Ultrasound system requires experienced therapists and adequate‐quality images. Images are considered adequate in 73% to 95% of patients.^(^ ^6^ ^–^ ^8^ ^)^ Poor images are typically attributed to inadequate bladder filling, larger abdominal girth, and the relative position of the prostate in relationship to the pubic arch.^(7)^ Even when images are deemed adequate, alignments by the therapists can be off by more than 5 mm in 3% of patients.^(6)^

To further improve treatment delivery technique, we use a CT in the linear accelerator radiation treatment delivery room to localize and correct for target position changes in a manner analogous to that used in the BAT Ultrasound system. The present paper describes the first part of a prospective trial that compared the isocenter shifts produced by the BAT Ultrasound system with the shifts produced by software provided by Siemens for the Primatom CT‐on‐Rails (Siemens Medical Solutions, Concord, CA).

## II. PATIENTS AND METHODS

The study included 15 consecutive patients with localized prostate cancer. All patients signed our institutional review board–approved informed consent. Patients were simulated in an Alpha Cradle cast in the supine position. Simulations using CT and magnetic resonance were performed and fused as previously described.^(9)^

All patients were treated with intensity‐modulated radiation therapy (IMRT) to doses between 74 Gy and 76 Gy, based on their respective risk stratifications.^(10)^ Inverse treatment planning was carried out using the Corvus treatment planning system (Nomos). Currently, it is the institution's policy for patients to undergo BAT Ultrasound^(11)^ before each treatment to reduce daily setup error. The BAT Ultrasound system works by obtaining coordination of the x, y, and z directions in comparison with the simulation isocenter. Like the BAT Ultrasound, CT‐on‐Rails also obtains x, y, and z coordinates.

For the present study, CT scans were obtained twice weekly in the treatment room, using the Primatom CT‐on‐Rails system. Our analysis includes 218 alignments from the 15 separate patients. All alignments were performed by one of four radiation therapists. Each therapist has several years' experience with the BAT system and also underwent initial training with the Volume Targeting prototype software (Siemens Medical Solutions). The initial training consisted of 20 – 30 alignments under the guidance of a physicist or physician on a previous set of data from patients who underwent multiple CT simulations.^(3)^

As is routine at our institution, ultrasound alignments were checked daily by the treating physician following treatment. For any shift greater than 1 cm, a physicist or a physician reviewed alignments while the patient was on the table. A similar approach was used when obtaining the CT in the treatment room. All CT alignments were reviewed by a physicist or physician within 1 week. If a significant disagreement (>10 mm) was observed between the ultrasound and CT alignments, the CT was reviewed following treatment.

Instances of disagreement were infrequent, occurring in fewer than 3% of alignments. Most of the large discrepancies were related to the learning curve of the therapists in trying to determine the prostate–bladder interface on a reconstructed CT with a 3‐mm slice thickness. In the sagittal plane, the isocenter shifts for all CT alignments were calculated with the Volume Targeting prototype software. That prototype software is analogous to the BAT Ultrasound.

### A. Daily shift calculation

**Figure 1:**
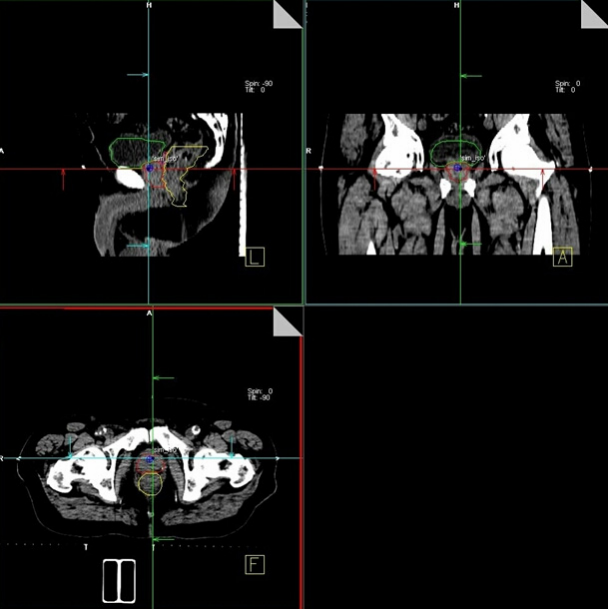
Three‐dimensional alignment of the simulation contours with the anatomy in the pre‐treatment image. Primatom CT‐on‐Rails system (Siemens Medical Solutions, Concord, CA), shown in the figure, and B‐mode Acquisition and Targeting (BAT) Ultrasound system (Nomos, Sewickley, PA) have a similar alignment interface. Primatom systems require the operator to use radio‐opaque markers to define the machine isocenter in the pre‐treatment scan (as shown in the figure).

The two systems (BAT Ultrasound and CT‐on‐Rails) work in similar fashion: The user aligns the contours from the CT simulation with the CT or ultrasound image. The system then calculates the distance between the planning isocenter (ideal position of the machine isocenter inside the patient's body) and the actual location of the machine isocenter before the alignment (that is, the isocenter shift; Fig. 1).

The contours used for the alignment were the prostate, bladder, rectum, and proximal seminal vesicles. The seminal vesicles were outlined as two separate structures: proximal and distal seminal vesicles. The proximal seminal vesicles include the first 9 mm. Only the proximal vesicles were used for alignment, because the distal seminal vesicles do not always move with the prostate.

The landmarks used for matching images were the proximal seminal vesicles and the prostate‐bladder interface in the superior‐inferior direction (sagittal images); the shape of the prostate and the prostate‐rectum interface were used in the AP and lateral direction alignments respectively.

Once obtained, the contours were transferred from the virtual simulation computer to each of the systems via a digital imaging and communication in medicine radiotherapy (DICOM‐RT) connection. A DICOM RT connection was also used to transfer the planning isocenter to the BAT system. In the Volume Targeting system, the planning isocenter was created manually with a precision of 0.1 mm using its CT coordinates as obtained from the virtual simulation station. Any discrepancies in the results related to differences in contours (used for alignment) or differences in the position of the planning isocenter were therefore eliminated.

The two systems use different approaches to define the actual machine isocenter in the patient's body before the shift. The ultrasound transducer from the BAT system is attached to a robotic arm, calibrated in the treatment room with precision of better than 1 mm. The relative location of the acquired ultrasound images with respect to the machine isocenter is therefore automatic. In contrast, the daily localization CT scans are related to the room isocenter using three radio‐opaque markers (BBs). The markers are placed on the patient's skin at the points identified by the room lasers (patient left, right, and anterior) before the scan. The treatment table is rotated 180 degrees (scanning position) and the localization CT scan is obtained. The table is then rotated back to 0 degrees, and the positions of the markers with respect to the lasers are re‐examined to ensure that the markers have not moved. The Volume Targeting prototype software is then used to manually calculate the shift based on the three BBs. The position of each BB is manually located. The software then generates the coordinate shift in comparison with the planning isocenter.

All CT scans had a slice thickness of 3 mm. The software performs multi‐plane reconstruction leading to a 1 ‐mm resolution in the superior‐inferior direction.

### B. Statistical analysis

#### *B.1 Comparison of CT versus ultrasound*

Isocenter shifts were recorded separately for the CT and the ultrasound. A correlated *t*‐test was used to evaluate the two sets for statistically significant differences. The correlation coefficients of the two sets of isocenter shifts were calculated. Statistical analyses were performed separately for each axis (that is, AP, right‐left, superior‐inferior).

The difference between the shifts (BAT−CT) was determined for each observation. Consider two variables *B* (the shifts obtained by the ultrasound) and *C* (the shifts obtained by the CT‐on‐Rails), with standard deviations $\sigma_{B}$ and $\sigma_{C}$ and mean values $\bar{B}$ and $\bar{C}$ respectively. The standard deviation and mean value of the difference A=B−C are given as

(1) $$\bar{A}=\bar{B}-\bar{C},\;\text{and}$$

(2) $$\sigma_{A}^{2}=\sigma_{B}^{2}+\sigma_{C}^{2}.$$

The standard deviation and difference of *A* is measured from the acquired data, and the standard deviation of *C* is estimated (as described in subsection A). The standard deviation of *B* can thus be calculated using equation 2.

#### *B.2 Assessment of the random uncertainty (standard deviation) of the CT shifts*

The two main sources of random error in the alignments of the CT are

- alignment of the prostate contour from the simulation scan to the localization scan, and
- identification of the machine isocenter in the localization scan using the BBs.

Therefore, the standard deviation of the total random error is

(3) $$\sigma_{C}^{2}=\sigma_{BB}^{2}+\sigma_{match}^{2},$$

where $\sigma_{\text{BB}}$ 1s attributable to the random error in identifying the BBs (which is multifactorial, because of breathing, intrafraction motion, and identification of the BB), and $\sigma_{\text{match}}$ is attributable to error in the matching of the contours.

The distribution of errors in the match can be evaluated by performing multiple shift calculations using the same localization scan (inter‐ and intra‐user variability of the results). Because the localization scan is the same, the random error attributable to the BBs is zero. If the BBs are placed incorrectly, the result will be a systematic error in the series of observations, but not a random one.

**Figure 2:**
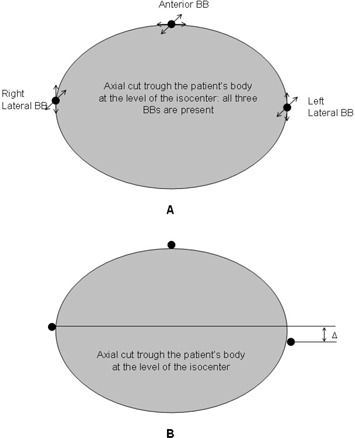
Mathematical model used to estimate random errors in isocenter coordinates because of motion of the radio‐opaque markers (BBs) during the scan. (A) Two degrees of freedom are used for every BB. (B) The standard deviation of the motion of a single BB in a given direction is calculated based on the standard deviation of the distance in anterior– posterior (AP) direction between the two lateral BBs.

The error attributable to identifying the BB can be evaluated by a simple model assuming that each BB can move randomly and independently in two directions [Fig. 2(a)]. For example, the lateral BBs can move in the AP and superior‐inferior direction, and the anterior BBs can move in the lateral and superior‐inferior directions. These random errors are assumed to have the same standard deviation σ.

The position of the isocenter in the longitudinal direction is defined as an average superior‐inferior position of all three BBs. The AP position is defined by the two lateral BBs, and the lateral position, only by the anterior BB. Therefore the $\sigma_{\text{BB}}$ will be different in the different directions, and it will be related to *o* (standard deviation of a single BB) as follows:

(4) $$\sigma_{BB,LAT}=\sigma ,$$

(5) $$\sigma_{BB,AP}=\frac{\sigma }{\sqrt{2}},\;\text{and}$$

(6) $$\sigma_{BB,LONG}=\sqrt{\frac{\sigma^{2}}{3}+{\left(\frac{s}{4}\right)}^{2}}\cdot$$

In the longitudinal direction, the standard deviation is adjusted for the slice thickness artifact, *s*, assuming additional random error of one fourth of the slice thickness (1 standard deviation). The maximum error is one half the slice thickness, and using a Gaussian distribution, it was assumed to equal 2 standard deviations. Therefore, 1 standard deviation is equal to one half of s/2—that is, s/4.

In our study, s=3 mm. This adjustment was not needed in the AP and lateral directions, because the voxel size is smaller than 1 mm, and the correction would be minor as compared with the motion of the radio‐opaque markers.

The standard deviation of each BB is calculated through a series of measurements of the distance between the two lateral BBs (Δ) in the AP direction [Fig. 2(b)]. Because the BBs move independently, the relationship between σ and $\sigma_{\Delta }$ will be

(7) $$\sigma =\frac{\sigma_{\Delta }}{\sqrt{2}}\cdot$$

The measurements must be performed using different localization scans. Then, equations 4, 5, and 6, in combination with equation 7, will give the standard deviation of the random error of the isocenter position. Further, if the values of $\sigma_{\text{BB}}$ and $\sigma_{\text{match}}$ are plugged into equation 3, the result is the total standard deviation of the random error of the CT shifts.

## III. RESULTS

### A. Comparison of CT‐on‐Rails and BAT Ultrasound

**Figure 3:**
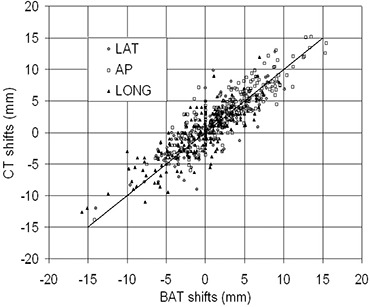
System‐versus‐system shifts for the B‐mode Acquisition and Targeting (BAT) Ultrasound system (Nomos, Sewickley, PA) and the Primatom CT‐on‐Rails system (Siemens Medical Solutions, Concord, CA). Data for 218 alignments are presented. The solid line is the line of perfect agreement between the two systems. The correlation coefficients are 0.877 [anterior‐posterior (AP)], 0.842 (lateral), and 0.831 (longitudinal).

Fig. 3 demonstrates the correlation between the isocenter shifts derived from the CT and the ultrasound. A high level of correlation is evident between the CT‐derived and the ultrasound‐derived isocenter shifts (correlation coefficients: 0.877 AP, 0.842 lateral, and 0.831 superior–inferior).

The differences between the CT and the BAT shifts were separated into systematic differences (average value of all differences) and random differences (standard deviations). The systematic differences (Table 1) were −0.62 mm (AP), −0.20 mm (lateral), and −0.32 mm (superior–inferior), and the random differences were 2.16 mm (AP), 2.14 mm (lateral), and 2.36 mm (superior–inferior). The correlated *t*‐tests (Table 2) demonstrated a statistically significant systematic difference between the two sets of data in the AP $\left(p=3.7\times 10^{-5}\right)$ and superior–inferior directions (p=0.048), but not in the lateral direction (p=0.175).

**Table 1:** Statistical analysis of the differences between the two sets of shifts

	Anterior—posterior	Lateral	Longitudinal
Average/mean (mm)	−0.62	−0.20	−0.32
Standard deviation (mm)	2.16	2.14	2.36
Average absolute (mm)	1.78	1.60	1.86
Group mean [mean of individual means (mm)]	−0.61	−0.20	−0.30
Standard deviation of individual means (mm)	0.93	0.56	1.01
Group mean of the standard deviations [RMS of individual standard deviations (mm)]	2.02	2.13	2.21

**Table 2:** Comparison between the two sets of shifts (computed tomography and ultrasound): correlation coefficients and p values from the correlated t‐test

	Correlation coefficient	Correlated *t*‐test (*p* values)
Anterior–posterior	0.877	$3.7\times 10^{-5}$
Lateral	0.842	0.175
Longitudinal	0.831	0.048

**Figure 4:**
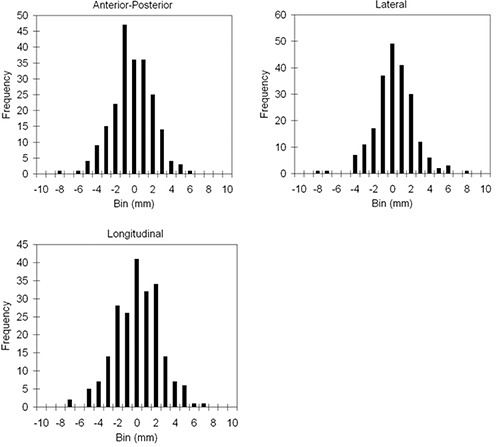
Histogram of the differences between the B‐mode Acquisition and Targeting (BAT) Ultrasound (Nomos, Sewickley, PA) and Primatom CT‐on‐Rails (Siemens Medical Solutions, Concord, CA) shifts. The size of the bin is 1 mm. The results are presented separately in the three directions.

Fig. 4 demonstrates the distribution of the differences between the CT and ultrasound shifts in all three directions. The symmetry of the distributions demonstrates the small systematic errors. The distribution in the longitudinal direction is wider, consistent with the larger standard deviation. The Shapiro–Wilkins test was used to test the distributions of the differences for non‐normality. The results showed that, in both the AP and longitudinal directions, the distributions were normal: p=0.846 (AP) and p=0.862 (superior–inferior). In the lateral direction, the test demonstrated a statistically significant non‐normality (p=0.001).

Table 3 shows the average and corresponding standard deviations between ultrasound and CT for each individual patient. The maximum average values are in the order of 2 mm in the AP and superior–inferior directions and less than 1 mm in the lateral direction. These values are based on 13 – 15 observations per patient, leading to relatively high standard errors of the mean value (defined as the standard deviation divided by the square root of the number of observations from Table 3) of the parent distribution.

**Table 3:** Systematic differences (average) and random differences (standard deviations) between ultrasound and computed tomography alignments

	Anterior–posterior [mm (SD)]	Lateral [mm (SD)]	Longitudinal [mm (SD)]
Patient 1	−0.78	0.52	−0.52
	(0.91)	(2.50)	(1.92)
Patient 2	0.69	−0.61	0.75
	(2.9)	(1.64)	(1.50)
Patient 3	0.38	0.21	−0.67
	(2.19)	(2.26)	(2.18)
Patient 4	0.21	−0.16	0.37
	(1.55)	(1.64)	(3.00)
Patient 5	−0.81	−1.28	1.32
	(2.20)	(1.32)	(2.09)
Patient 6	0.3	−0.47	1.11
	(2.05)	(1.46)	(1.36)
Patient 7	0.04	0.14	−0.37
	(1.82)	(1.59)	(2.36)
Patient 8	−0.87	−0.44	1.09
	(1.90)	(2.86)	(2.33)
Patient 9	−1.02	−0.92	−0.64
	(1.45)	(1.86)	(1.92)
Patient 10	−1.48	0.66	−1.91
	(1.7)	(2.7)	(2.68)
Patient 11	−1.87	−0.21	−0.98
	(2.39)	(2.30)	(2.08)
Patient 12	−1.14	0.33	−0.51
	(2.35)	(2.33)	(2.02)
Patient 13	−1.4	0.25	−1.71
	(1.94)	(1.70)	(2.27)
Patient 14	−2.1	−0.85	−0.70
	(1.94)	(2.38)	(2.07)
Patient 15	0.71	−0.13	−1.12
	(2.23)	(2.60)	(2.79)

### B. Assessment of the random uncertainties in the CT shifts

The random error of the CT‐based alignment was estimated by the standard deviation of the shift results calculated 20 times for a given patient on a given day by an experienced user. This intra‐user variability statistic was carried out separately for 3 different patients. Patient X was a patient of small size with a lateral separation of 34 cm. Patient Y was a patient of average size (separation of 38.5 cm), and patient Z had a separation of 44 cm (Table 4). According to the results, an estimate of the standard deviations of the CT alignment for typical patients should fall into the following regions: 0.5 – 0.8 mm (AP), 0.5 – 0.8 mm (lateral), and 0.8 – 1.2 mm (longitudinal).

**Table 4:** Estimated random uncertainties (1 standard deviation) of the alignment of the planning contours with the localization computed tomography scan^a^

	Patient X (mm)	Patient Y (mm)	Patient Z (mm)
Anterior–posterior	0.47	0.80	0.58
Lateral	0.61	0.50	0.79
Longitudinal	0.80	0.89	1.18

a Results are based on an intra‐user variability study of an experienced user.

The random error in the machine isocenter definition was estimated separately for the same 3 patients. The statistics were based on 20 – 21 observations per patient, and the standard deviations of the isocenter coordinates were calculated using equations 4, 5, 6, and 7. Based on the results (Table 5), the estimated regions for standard deviations of the BBs are 0.5 – 0.6 mm (AP), 0.7 – 0.9 mm (lateral), and approximately 0.9 mm (longitudinal). Based on these 3 patients, with their varying abdominal girth, there appears to be no correlation between the uncertainties of the BBs and patient size. The BBs appear to move more in large patients with soft skin. However, the smallest patient in the study (patient X), had a problem holding his bladder, which also resulted in significant motion. Therefore, aside from skin motion, there may be multiple patient‐specific reasons for random motion of the BBs. Thus, we assumed that the uncertainties for a random patient may have arbitrary values within the stated regions.

**Table 5:** Estimated random errors (standard deviations) in the position of the machine isocenter inside the computed tomography–scanned volume, determined by radio‐opaque markers (BBs)

	Patient X	Patient Y	Patient Z
$\sigma_{\Delta }(\text{mm})$	1.30	1.04	1.17
σ(mm)	0.91	0.73	0.83
$\sigma_{\text{BB},\text{LAT}}(\text{mm})$	0.91	0.73	0.83
$\sigma_{\text{BB},\text{AP}}(\text{mm})$	0.64	0.52	0.58
$\sigma_{\text{BB},\text{LONG}}(\text{mm})$	0.92	0.86	0.89

LAT=lateral; AP=anterior−posterior; LONG=longitudinal (superior−inferior).

The total standard deviations of the CT‐based shifts, including errors from alignment and errors from BB motion alike, can be calculated based on the results in Tables 4 and 5 and in equation 3. Considering best‐ and worst‐case scenarios, the estimated ranges of the standard deviations for the total CT shifts are 0.7 – 1 mm (AP), 0.9 – 1.2 mm (lateral), 1.2 – 1.5 mm (superior–inferior).

## IV. DISCUSSION

The advantage of CT over an ultrasound system is that the target is compared using the same modality as the simulation, which should make training easier. More importantly, tumor sites other than prostate cancer will benefit from precise localization. Based on the results presented here, localization of the prostate has a total uncertainty (2 standard‐deviations interval) of 2 – 3 mm when using the Primatom CT‐on‐rails system and of approximately 4 mm when using the BAT Ultrasound image. Furthermore, concern about displacement of the prostate by the ultrasound^(^ ^12^ ^,^ ^13^ ^)^ and the discomfort of treatment with a full bladder can lead to difficulties with this approach.

The systematic differences between the CT and the ultrasound alignments were very low, and they were not statistically significant in the lateral direction. Statistically significant differences arose in systematic differences between the ultrasound and the CT in the AP and superior–inferior directions, but with values less than 1 mm, this result is not clinically relevant.

Previous studies have reported considerable systematic errors, especially in the superior– inferior direction^(^ ^14^ ^,^ ^15^ ^)^. One reason for the low systematic error in the present study is the use of seminal vesicles as a landmark for superior–inferior alignment. The systematic errors of the two modalities (CT and ultrasound) were not estimated separately in this work. Therefore, it is possible that large systematic errors for each modality could occur, but the systematic difference between them would be small only if the systematic differences of the two modalities are similar (see Table 1). The ranges of standard deviations for the BAT shifts were 1.9 – 2.0 mm in the AP direction, 1.8 – 2.0 mm in the lateral direction, and 1.9 – 2.1 mm in the superior– inferior direction.

It is essential to point out that these numbers do not characterize the equipment itself, but rather the whole method. For instance, use of the proximal seminal vesicles as a landmark for superior–inferior alignment is very helpful for both the ultrasound and CT alignment.

In regard to the BAT Ultrasound system, another important technique is the use of a real‐time three‐dimensional alignment before the single‐plane images are captured. In other words, the CT and the ultrasound alignments lead to the same systematic error in every direction, but that result is very unlikely, because different imaging modalities are used with differing isocenter definition techniques. The only common element between the workflows of the two systems is the set of contours transferred from the simulation scan. It is true that the contours are not perfect, which can lead to a discrepancy. However, the goal of localization alignment in radiation therapy is to align the target in the correct position with the dose cloud, and the dose cloud in IMRT planning is always delivered to the contour, not to the actual structure (if discrepancy arises between these two). Therefore, what is really important is to place the anatomy of the target, as identified in the pre‐treatment image, within the target contour used for dose calculation.

## V. CONCLUSIONS

Isocenter‐derived shifts obtained by a CT in the treatment room were rhighly correlated with ultrasound‐derived shifts. Daily CT localization can therefore be used for other disease sites not appreciably visualized by ultrasound.
